# Genetic profiles of Barrett’s esophagus and esophageal adenocarcinoma in Japanese patients

**DOI:** 10.1038/s41598-021-97249-9

**Published:** 2021-09-03

**Authors:** Mamoru Tokunaga, Kenichiro Okimoto, Naoki Akizue, Kentaro Ishikawa, Yosuke Hirotsu, Kenji Amemiya, Masayuki Ota, Keisuke Matsusaka, Motoi Nishimura, Kazuyuki Matsushita, Tsubasa Ishikawa, Ariki Nagashima, Wataru Shiratori, Tatsuya Kaneko, Hirotaka Oura, Kengo Kanayama, Yuki Ohta, Takashi Taida, Keiko Saito, Tomoaki Matsumura, Tetsuhiro Chiba, Hitoshi Mochizuki, Makoto Arai, Jun Kato, Jun-ichiro Ikeda, Masao Omata, Naoya Kato

**Affiliations:** 1 Department of Gastroenterology, Graduate School of Medicine, Chiba University, Inohana 1-8-1, Chiba, 260-8670 Japan; 2 Genome Analysis Center, Yamanashi Prefectural Central Hospital, Yamanashi, Japan; 3 Department of Molecular Pathology, Graduate School of Medicine, Chiba University, Chiba, Japan; 4 Division of Laboratory Medicine, Chiba University Hospital, Chiba, Japan; 5 Department of Medicine and Clinical Oncology, Graduate School of Medicine, Chiba University, Chiba, Japan; 6 The University of Tokyo, Tokyo, Japan

**Keywords:** Cancer, Genetics, Gastroenterology

## Abstract

The genetic characteristics of Barrett’s esophagus (BE) and esophageal adenocarcinoma (EAC) in the Japanese population is unclear. This study aims to investigate the genetic characteristics from nondysplastic BE (NDBE) to early EAC in Japan. Clinical information was collected. Moreover, the genetic profile of NDBE without concurrent dysplasia, early EAC, and surrounding BE were also investigated using endoscopic biopsy samples and formalin-fixed, paraffin-embedded specimens from Japanese patients by targeted next-generation sequencing. Immunohistochemical staining for p53 was also performed for EAC lesions. Targeted NGS was performed for 33 cases with 77 specimens. No significant difference exists in the NDBE group between the number of putative drivers per lesion in the short-segment Barrett’s esophagus (SSBE) and long-segment Barrett’s esophagus (LSBE) [0 (range, 0–1) vs. 0 (range, 0–1). *p* = 1.00]. *TP53* putative drivers were found in two patients (16.7%) with nondysplastic SSBE. *TP53* was the majority of putative drivers in both BE adjacent to EAC and EAC, accounting for 66.7% and 66.7%, respectively. More putative drivers per lesion were found in the EAC than in the NDBE group [1 (range, 0–3) vs. 0 (range, 0–1). *p* < 0.01]. The genetic variants of *TP53* in the Japanese early EAC were similar to those in western countries. However, *TP53* putative drivers were detected even in Japanese patients with nondysplastic SSBE. This is significant because such nondysplastic SSBE might have higher risk of progressing to high-grade dysplasia or EAC. The risks of progression may not be underestimated and appropriate follow-ups may be necessary even in patients with SSBE.

**Trial registration:** This study was registered at the University Hospital Medical Information Network (UMIN000034247).

## Introduction

Barrett’s esophagus (BE) is pathologically defined as a columnar-lined epithelium (CLE) that replaces the squamous epithelium of the esophagogastric junction (EGJ) during the healing process from esophagitis^1,2^. In addition, it is a precursor of esophageal adenocarcinoma (EAC)^3^. In western countries, EAC accounts for approximately 60% of all esophageal cancers and is considered an important disease due to its poor prognosis^4–6^. Appropriate endoscopic surveillance is required to detect EAC at an early stage. However, diagnosing early-stage EAC arising from an inflamed BE is still challenging^7,8^.

Recently, some genetic studies have been conducted to predict the development or concurrent dysplasia using clinical specimens from EAC and BE mucosa. With the advent of next-generation sequencing (NGS), several somatic mutations, represented by *TP53*, have been reported to be associated with EAC^9–16^.

The number of patients with EAC has been increasing in Japan^17,18^. However, it is unclear what genetic alterations underlie early EAC and adjacent BE in Japanese patients. Thus, this study aimed to investigate the genetic and clinical characteristics of the pathway from nondysplastic BE (NDBE) to early EAC in Japan using endoscopic specimens from Japanese patients with NDBE and early EAC.

## Methods

### Study design and patients

This study examined patients with NDBE and with early-stage EAC based on an esophagogastroduodenoscopy at Chiba University Hospital (Chiba, Japan) between November 2017 and March 2020. With regard to patients with NDBE, 12 with short-segment Barrett’s esophagus (SSBE) and 12 with long-segment Barrett’s esophagus (LSBE) were enrolled in this study. For patients with the SSBE, those with atrophic gastritis were precluded from the study to refrain from falsely diagnosing BE. Eleven lesions from nine patients who underwent endoscopic mucosal resection with cap (EMR-C) and endoscopic submucosal resection (ESD) were also examined for patients with early-stage EAC. To achieve en-bloc resection, we generally perform ESD for EAC 10 mm ≤ . We generally perform EMR-C for EAC 10 mm > .

Detailed clinical information from all patients, including height, body weight, smoking and drinking history, medication, colorectal tumor history, EAC history, recurrence of EAC, and overall survival. *Helicobacter pylori* eradication history were obtained. *H. pylori* infection status was confirmed by serum IgG antibody against *H. pylori* (E Plate Eiken *H. pylori* antibody. Eiken Chemical Co., Ltd., Tokyo, Japan).

This study was approved by the Bioethics Committee of Chiba University Hospital (UMIN000034247). Written informed consent was obtained from all patients in this study. all methods were performed in accordance with the relevant guidelines and regulations.

### Esophagogastroduodenoscopy

Esophagogastroduodenoscopy was conducted using the LASEREO VP-7000 system with an EG-L600WR7 or EG-L600ZW7 endoscope (FUJIFILM, Tokyo, Japan). The EVIS LUCERA ELITE CV-290 system with a GIF-H260Z or GIF-H290T endoscope (Olympus, Tokyo, Japan) was also used. Moreover, CLE is defined as the area from the squamous-columnar junction (SCJ) to the lower end of the palisade vessels (PVs) when the PVs are recognized or the area from the SCJ to the upper end of the gastric folds when the PVs are not recognized. This study defined CLE ≥ 1 cm as BE with or without intestinal metaplasia. CLE ≥ 3 cm in maximum length was classified as LSBE. CLE < 3 cm was classified as SSBE. The Plague and Paris classifications were used to indicate BE length^19^ and classify EAC^20^, respectively. Reflux esophagitis was evaluated and graded according to the Los Angeles classification (A–D)^21^. The degree of atrophic gastritis was evaluated according to the Kimura–Takemoto classification (closed or open type)^22^.

### Endoscopic sample collection

**Figure 1:**
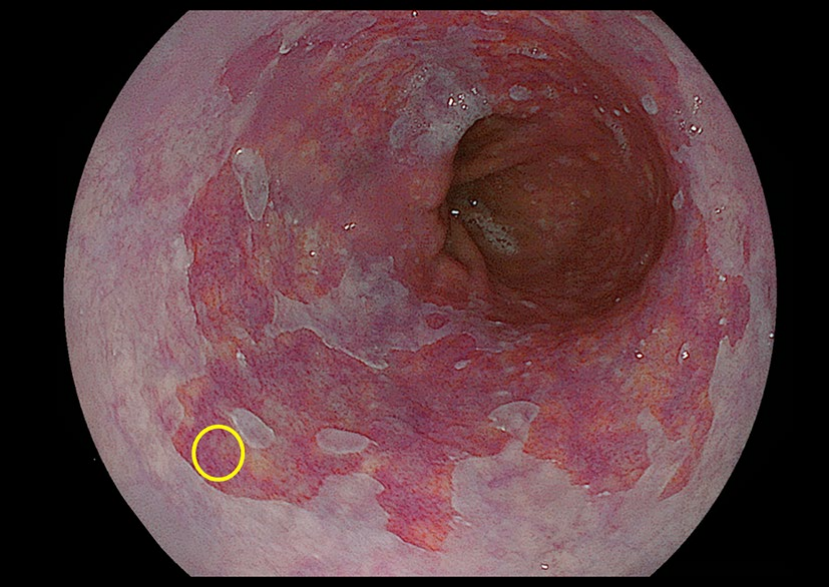
Representative case of targeted biopsy for nondysplastic Barrett’s esophagus (C4M6, long-segment Barrett’s esophagus case 8). Biopsy specimens were taken from the most elongated part of the Barrett’s esophagus to separate from the epithelium of the stomach. The *yellow circle* shows the area where the biopsy was taken.

Endoscopic biopsy specimens were used for NDBEs using large-capacity forceps (Radial Jaw 4, Boston Scientific, Marlborough, MA, USA). This device can obtain 5 mm tissue. Biopsy specimens were taken from the most elongated part of the BE to separate from the CLE of the stomach (Fig. 1). Two biopsies were performed from the same region. one was evaluated pathologically and the other for genetic evaluation. Endoscopically resected and formalin-fixed, paraffin-embedded (FFPE) specimens were used for early-stage EACs.

### Pathological analysis

The pathological evaluation was carried out using hematoxylin and eosin-stained sections by two pathologists (KM and MO) in our institution. The presence of carcinoma/dysplasia were evaluated for all patients, and whether the lesion was EAC arising from BE was confirmed for patients with EAC according to the 11th Japanese classification^23^. All EAC specimens were also immunostained by the anti-p53 antibody. Formalin-fixed paraffin-embedded samples were thin-sliced at 4 µm. The sections were then deparaffinized before the staining procedure. Monoclonal mouse antihuman p53 protein was used as the primary antibody for immunohistochemistry (Clone DO-7, Agilent, CA, USA). The slides were incubated at room temperature for 20 min with the primary antibody. Each EAC case was classified into three subtypes as immunostaining pattern of p53 (overexpression-type mutation, null cell-type mutation, and wild-type patterns).

### Deoxyribonucleic acid extraction

DNA was extracted from each tissue as shown in the supplementary methods.

### Esophageal cancer panel

**Figure 2:**
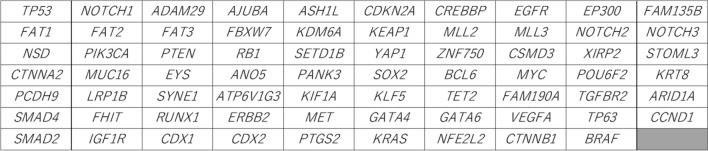
List of 69 significant mutated genes included in the panel. Each box shows each gene included in the panel.

The in-house panel was designed by referring to the previous studies with Ion AmpliSeq designer software (Thermo Fisher Scientific)^24,25^. Sixty-nine significantly mutated genes (SMGs) were included to cover SMGs for both EAC and esophageal squamous cell carcinoma (Fig. 2). The SMGs were selected according to (1) genes that are often involved in esophageal cancer (EC), obtained from TCGA and other projects^9,10,26–28^ and (2) genes frequently mutated in EC, referring to the COSMIC database (http://cancer.sanger.ac.uk/cancergenome/projects/cosmic). Finally, the panel consisted of 4410 primer pairs.

### Targeted NGS

Targeted NGS was carried out using the panel as shown in the supplementary methods.

### NGS data analysis

NGS data was analyzed as shown in the supplementary methods. Buffy coat DNA was used as a reference to identify variants in EAC and NDBE. For each identified mutant gene, we analyzed whether it was oncogenic or not concerning OncoKB (https://www.oncokb.org/)^29^. In this study, oncogenic and likely oncogenic mutations were defined as putative drivers.

### Statistical analysis

The patients’ characteristics were analyzed using Fisher’s exact test, and the number of putative drivers per lesion in each group was analyzed using the Mann–Whitney *U* test. In addition, obesity, smoking, and other factors, known as EAC risk, were also analyzed to make a difference in putative drivers per lesion using the Mann–Whitney *U* test. All statistical analyses were performed using SPSS 26.0 (SPSS Inc., Chicago, IL, USA), and a *p* value < 0.05 was considered to be statistically significant.

## Results

### Patients’ characteristics and endoscopic findings

**Figure 3:**
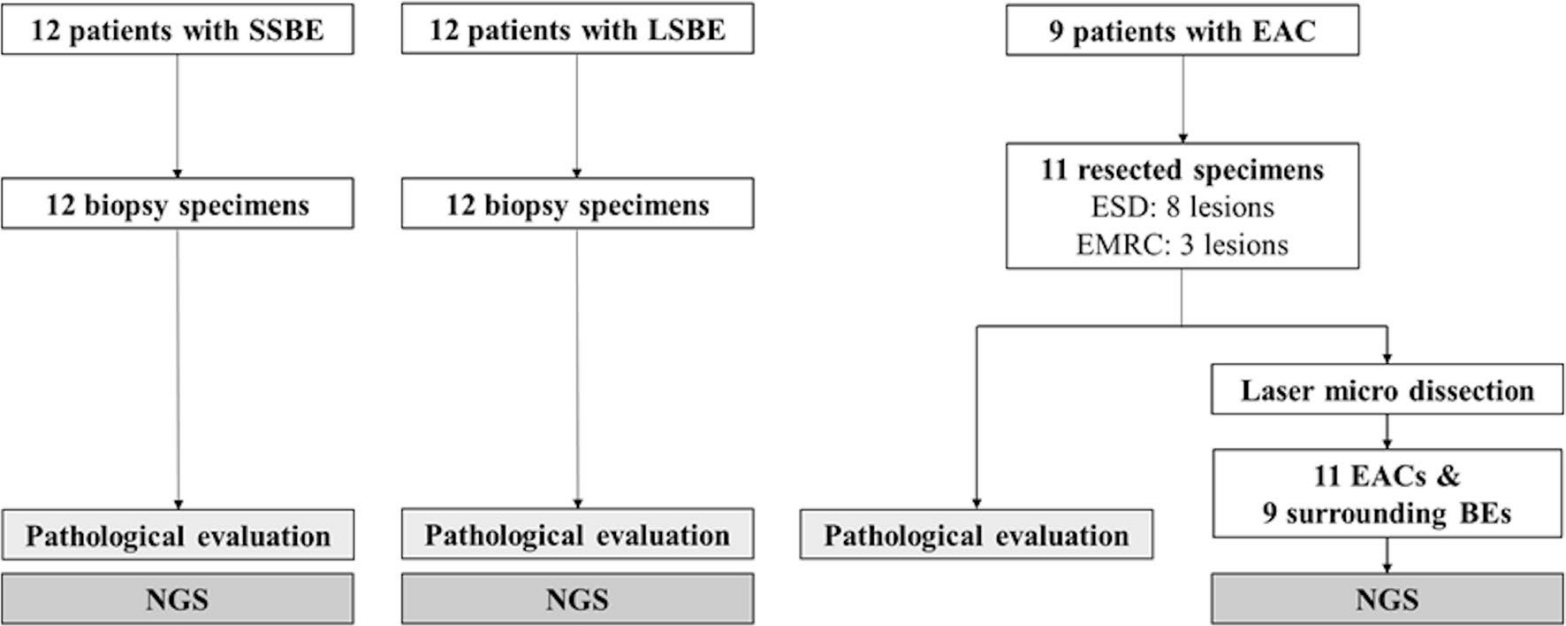
Study flow. *EAC* esophageal adenocarcinoma, *EMR-C* endoscopic mucosal resection with cap, *ESD* endoscopic submucosal dissection, *LSBE* long-segment Barrett’s esophagus, *SSBE* short-segment Barrett’s esophagus.

Figure 3 and Table 1 show the study flow and patients’ characteristics and endoscopic findings, respectively. Twenty-four patients with NDBE (12 with SSBE and 12 with LSBE) and nine patients with EAC were included in this study. The median age of the patients at biopsy or initial endoscopic resection was 66 (range, 22–87) years. In addition, more male patients (25 males and eight females) were in this study. The EAC group had a significantly higher percentage of drinkers than the NDBE group (88.9% vs. 45.8%. *p* = 0.047), and no statistically significant differences exist in other patients’ characteristics. In patients with EAC, one patient was treated endoscopically for one lesion. A year later, two lesions were found to have recurred and were endoscopically resected. All the other patients underwent endoscopic resection for one lesion. ESD and EMR-C (eight and three lesions, respectively) were the tissue-sampling methods. The median BE length and median lesion size were 1.5 cm (range, 1–4) and 10 mm (range, 2–27), respectively. The numbers of 0-IIa, 0-IIb, and 0-IIc were five (45.5%), five (45.5%), and one (9.1%), respectively (Table 2).

**Table 1:** Patients’ characteristics.

	SSBE	LSBE	EAC
Number of patients	12	12	9
Age, median (range)	62 (50–78)	65 (22–80)	69 (58–87)
Sex (male/female)	7/5	10/2	8/1
BMI (kg/m^2^), median (range)	23.4 (20.2–30.0)	24.3 (17.3–29.0)	24.7 (21.6–28.1)
Smoking (+/−)	5/7	5/7	7/2
Drinking (+/−)	5/7	6/6	8/1
Amount of drinking (g/day), median (range)	0 (0–60.0)	1 (0–57.5)	31.9 (0–57.2)
**Medicine (+/−)**			
Statin	6/6	2/10	2/7
PPI or PCAB	7/5	7/5	2/7
H2RA	0/12	1/11	1/8
NSAIDs	1/11	0/12	0/9
Aspirin	0/12	1/11	0/9
**Medical history (+/−)**			
Colorectal tumor	1/11	1/11	3/6
EAC	0/12	0/12	1/8
*H. pylori* infection (+/−/unknown)	0/10/2	0/9/3	1/8/0
**Endoscopic findings**			
Hiatal hernia (+ −)	9/3	10/2	6/3
RE (none/A/B/C/D)^a^	8/4/0/0/0	11/1/0/0/0	6/1/1/1/0
Length of CLE (cm), median (range)	1 (1–2)	5 (3–17)	1.5 (1–4)
Gastric atrophy (none/closed type/open type)^b^	12/0/0	10/2/0	6/2/1

*BE* Barrett’s esophagus, *BMI* body mass index, *CLE* columnar-lined epithelium, *EAC* esophageal adenocarcinoma, *GERD* gastroesophageal reflux disease, *H2RA* H2 receptor antagonist, *LSBE* long-segment Barrett’s esophagus, *NSAIDs* nonsteroidal anti-inflammatory drugs, *PCAB* potassium-competitive acid blocker, *PPI* proton pump inhibitor, *SSBE* short-segment Barrett’s esophagus, *RE* reflux esophagitis.

^a^Reflux esophagitis was graded according to the Los Angeles classification (A–D).

^b^Gastric atrophy was graded according to the Kimura–Takemoto classification (closed or open type).

**Table 2:** Endoscopic and pathological findings in esophageal adenocarcinoma.

	EAC
Number of patients	9
Number of lesions	11
Diameter, median (range)	10 (2–27)
**Paris classification**	
0-IIa, *n* (%)	5 (45.5)
0-IIb, *n* (%)	5 (45.5)
0-IIc, *n* (%)	1 (9.1)
**Invasion depth**	
SMM, *n* (%)	9 (81.8)
LPM, *n* (%)	1 (9.1)
DMM, *n* (%)	1 (9.1)
**Immunohistochemical staining for p53**	
Overexpression-type mutation pattern, *n* (%)	8 (72.7)
Null cell-type mutation pattern, *n* (%)	1 (9.1)
Wild-type pattern, *n* (%)	2 (18.2)

*EAC* esophageal adenocarcinoma, *DMM* deep muscularis mucosae, *LPM* lamina propria mucosae, *SMM* superficial muscularis mucosae.

### Pathological findings

**Figure 4:**
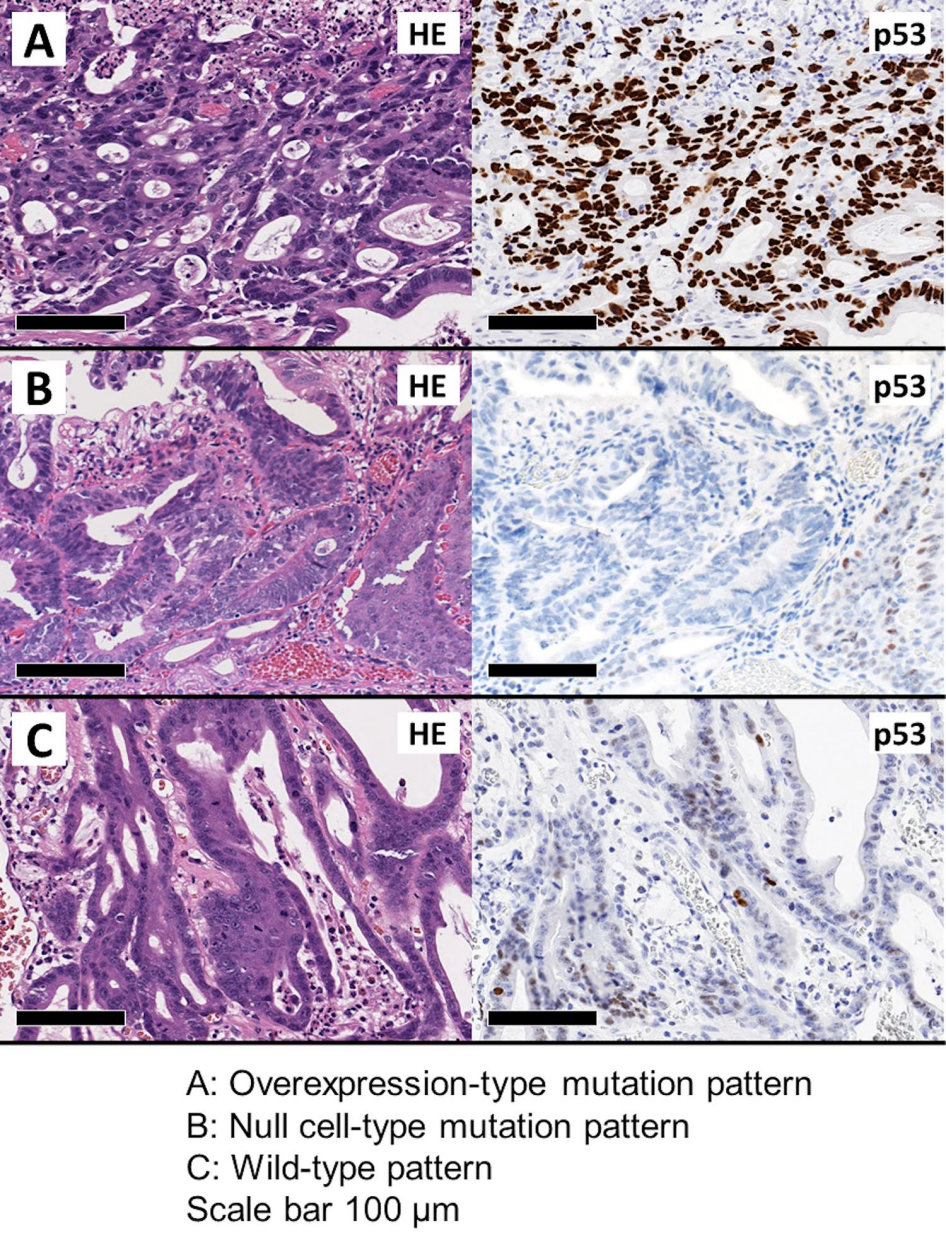
Experimental results for p53 staining. (**A**) Overexpression-type mutation pattern. (**B**) Null cell-type mutation pattern. (**C**) Wild-type pattern. Scale bar, 100 µm. *HE* hematoxylin and eosin.

The absence of dysplasia for NDBE patients was confirmed in the biopsy specimens. Moreover, all EAC lesions were confirmed to be EAC. Eight lesions (81.8%) were cancers in superficial muscularis mucosae (Table 2). The histological photographs of EACs stained by anti-p53 antibody are shown in Fig. 4. Among the 11 lesions, the numbers of overexpression-type mutation, null cell-type mutation, and wild-type patterns were eight (78.7%), one (9.1%), and two (18.2%), respectively (Table 2). In addition, three lesions from the same patient (EAC patient 1) were all overexpression-type mutation patterns.

### NGS analysis

**Figure 5:**
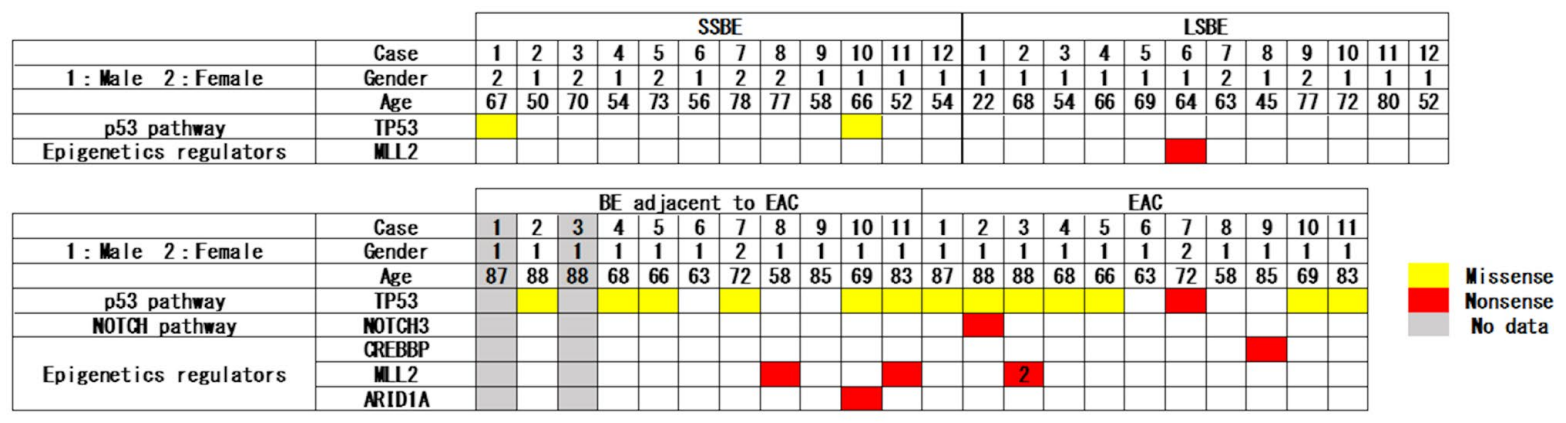
**G**ene plot showing putative drivers in each sample. *Putative drivers were judged with OncoKB* (https://www.oncokb.org/). *Yellow boxes* represent missense mutations. *Red boxes* represent nonsense mutations. Each *Box without numbers* represents a single mutation. The *numbers in the boxes* indicate the number of mutations. *BE* Barrett’s esophagus, *EAC* esophageal adenocarcinoma, *LSBE* long-segment Barrett’s esophagus, *SSBE* short-segment Barrett’s esophagus.

Targeted NGS was performed for 33 cases with 77 specimens. SSBE biopsy specimens, LSBE biopsy specimens, laser microdissected BE adjacent to EAC specimens, laser microdissected EAC specimens, and buffy coats from each patient were 12, 12, 9, 11, and 33, respectively. Consequently, 24 putative drivers of five genes were found (Table 3 and Fig. 5).

**Table 3:** Differences in next-generation sequencing results between nondysplastic Barrett’s esophagus and esophageal adenocarcinoma.

	NDBE group		EAC group		*P* value		
	SSBE	LSBE	BE adjacent to EAC	EAC	SSBE vs. LSBE	BE adjacent to EAC vs. EAC	NDBE vs. BE adjacent to EAC
No. patients	12	12	9	9	N/A	N/A	N/A
No. samples	12	12	9	11	N/A	N/A	N/A
**NGS analysis**							
Putative drivers, *n*	2	1	9	12	N/A	N/A	N/A
Putative drivers per lesion, median (range)	0 (0–1)	0 (0–1)	1 (0–2)	1 (0–3)	1.00	1.00	< 0.01*
Coverage, median (range)	191 (147–234)	400	216 (147–431)	151 (108–613)	0.67	0.058	0.86
AF (%), median (range)	22.6 (3.4–41.9)	3.5	8.3 (6.1–22.2)	20.8 (5.4–58.9)	1.00	0.15	0.48
Missense, median (range)	0 (0–1)	0 (0–0)	1 (0–1)	1 (0–1)	0.51	0.94	0.01*
Nonsense, median (range)	0 (0–0)	0 (0–1)	0 (0–1)	0 (0–2)	0.76	0.82	0.21

*AF* Allele frequency, *BE* Barrett’s esophagus, *EAC* esophageal adenocarcinoma, *LSBE* long-segment Barrett’s esophagus, *NDBE* nondysplastic Barrett’s esophagus, *NGS* next-generation sequencing, *SSBE* short-segment Barrett’s esophagus, *N/A* not applicable.

*P < 0.05, Mann–Whitney *U* test.

### The mutational analysis in patients with NDBE

Three putative drivers of two genes were found in 24 patients (12 patients with SSBE and 12 patients with LSBE. Table 3 and Fig. 5). No significant difference exists between the number of putative drivers per lesion in the SSBE and LSBE groups [0 (range, 0–1) vs. 0 (range, 0–1). *p* = 1.00]. In the SSBE group, *TP53* putative drivers were found in two patients. One was an oncogenic mutation (coverage, 234. AF, 41.9%) and the other was likely oncogenic (coverage, 147. AF, 3.4%). In the LSBE group, *MLL2* nonsense mutation was found as a putative driver (coverage, 400. AF, 3.5%. Table 3 and Fig. 5).

### The mutational analysis in patients with EAC

Twenty-one putative drivers of five genes were found in nine patients with EAC (Table 3 and Fig. 5). No significant difference was found between the number of putative drivers per lesion in BE adjacent to EAC and EAC groups [1 (range, 0–2) vs. 1 (range, 0–3). *p* = 1.00]. In the BE adjacent to EAC group, nine putative drivers of three genes were found. Six of them were *TP53* putative drivers, and the coverage and AF (range) were 202 (147–382) and 11.7 (6.1–22.2), respectively. In the EAC group, 12 putative drivers of four genes were found. In addition, *TP53* putative drivers were found in eight cases, and the coverage and AF (range) were 182 (108–613) and 29.8 (10.2–58.9), respectively. Identical *TP53* putative drivers in tumor and nontumor areas were found in three cases. However, *TP53* putative drivers were found only in tumor areas in two cases. Thus, *TP53* putative drivers were found to differ between samples from EAC and BE adjacent to EAC even in the same patient (Table 4).

**Table 4:** Putative drivers of *TP53* in patients with esophageal adenocarcinoma.

Patient no	*TP53* mutation (allele frequency, coverage)	
	EAC	BE adjacent to EAC
1 (Lesion 1)	p.R280T (23.1%, 121)	N/A
1 (Lesion 2)	p.C135F (42.4%, 125)	p.R280T (7.8%, 167)
1 (Lesion 3)	p.R280T (10.2%, 108)	N/A
2	p.R280T (36.5%, 178)	p.R280T (14.8%, 243)
3	p.R280T (21.5%, 613)	p.R280T (22.2%, 216)
4	No mutation of *TP53*	No mutation of *TP53*
5	p.E204^a^ (39.8%, 186)	p.R280T (6.1%, 147)
6	No mutation of *TP53*	No mutation of *TP53*
7	No mutation of *TP53*	No mutation of *TP53*
8	p.R280T (20.2%, 243)	p.R280T (14.9%, 188)
9	p.C275G (58.9%, 224)	p.Q192H (8.6%, 382)

*BE* Barrett’s esophagus, *EAC* esophageal adenocarcinoma, *N/A* not applicable.

^a^Stop codon.

### The differences of mutations between patients with NDBE and EAC

More putative drivers per lesion were found in the EAC than the NDBE group (12 SSBE and 12 LSBE) [1 (range, 0–3) vs. 0 (range, 0–1). *p* < 0.01]. Similarly, more putative drivers per lesion were detected in the BE adjacent to EAC group than in the NDBE group [1 (range, 0–2) vs. 0 (range, 0–1). *p* < 0.01. Table 3]. In addition, more missense mutations were detected in the BE adjacent to EAC group than in the NDBE group [1 (range, 0–1) vs. 0 (range, 0–1). *p* = 0.01. Table 3].

### The association between mutations and the characteristics of patients

The association between the number of putative drivers in each of the 44 tissues (12 SSBE, 12 LSBE, 9 BE adjacent to EAC, and 11 EAC) and the characteristics of patients was assessed. Patients aged ≥ 65 years and those who drink an average of ≥ 30 g alcohol per day had more putative drivers per lesion (*p* < 0.01 and *p* < 0.01). When limited to patients with EAC, the difference in drinking habits was no longer observed (*p* = 1.00). However, patients aged ≥ 65 years had more putative drivers (*p* = 0.023). No statistically significant associations exist between the number of putative drivers per lesion and other clinical information, including gender, obesity, smoking history, hiatal hernia, and BE length (Table 5). Only one patient had recurrence of EAC (initial treatment 1 lesion and 2 lesions at recurrence, case1–3 in Fig. 5). The patient had *TP53* putative driver in all 3 lesions. *TP53* putative diver was identified in 5 out of other 8 lesions. The recurrent lesions had multiple putative driver mutation, while others did not. No patient died during observation period.

**Table 5:** Association between patients’ demographics and the number of putative drivers.

Clinical characteristics	Number of lesions, *n* (%)		Number of putative drivers per lesion, median (range)		*P* value
	Yes	No	Yes	No	
Male sex	35 (79.5)	9 (20.5)	0 (0–3)	0 (0–1)	0.47
Obesity (BMI ≥ 25)	14 (31.8)	30 (68.2)	0 (0–3)	0 (0–2)	0.49
Older age (≥ 65)	29 (65.9)	15 (34.1)	1 (0–3)	0 (0–1)	< 0.01*
Drinking habit (≥ 30 g per day)	18 (40.9)	26 (59.1)	1 (0–3)	0 (0–2)	< 0.01*
Smoking	26 (59.1)	18 (40.9)	1 (0–3)	0 (0–1)	0.055
Present *H. pylori* infection	10 (22.7)	34 (77.3)	0.5 (0–2)	0 (0–3)	0.95
Past history of colorectal tumor	7 (15.9)	37 (84.1)	1 (0–2)	0 (0–3)	0.18
**Medicine**					
PPI	15 (34.1)	29 (65.9)	0 (0–3)	0 (0–2)	0.53
PCAB	5 (11.4)	39 (88.6)	0 (0–3)	0.5 (0–2)	0.97
H2RA	3 (6.8)	41 (93.2)	0 (0)	0 (0–3)	0.73
NSAIDs	1 (2.3)	43 (97.7)	0 (0)	0 (0–3)	0.59
Aspirin	1 (2.3)	43 (97.7)	0 (0)	0 (0–3)	0.59
Statin	14 (31.8)	30 (68.2)	1 (0–3)	0 (0–2)	0.18
**Endoscopic findings**					
Hiatal hernia	33 (75.0)	11 (25.0)	0 (0–3)	0 (0–2)	0.69
RE (≥ Los Angeles grade A)	9 (20.5)	35 (79.5)	0 (0–1)	0 (0–3)	0.89
LSBE	18 (40.9)	26 (59.1)	0 (0–3)	0 (0–2)	0.77
Right anterior lesion	21 (47.7)	23 (52.3)	1 (0–2)	0 (0–3)	0.31
Atrophic gastritis	8 (18.2)	36 (81.8)	1 (0–2)	0 (0–3)	0.52

*BMI* body mass index, *GERD* gastroesophageal reflux disease, *H2RA* H2 receptor antagonist, *LSBE* long-segment Barrett’s esophagus, *NSAIDs* nonsteroidal anti-inflammatory drugs, *PCAB* potassium competitive acid blocker, *PPI* proton pump inhibitor, *RE* reflux esophagitis.

*P < 0.05, Mann–Whitney *U* test.

## Discussion

This study investigated the genetic profile of NDBE without concurrent dysplasia, early EAC, and the surrounding BE using endoscopic biopsy samples and FFPE specimens from Japanese patients. It is believed that no reports exist of genetic profile in Japanese NDBE, early EAC, and adjacent BE using endoscopic specimens including samples taken from laser capture microdissection (LCM) samples. This study is novel and has significance with the EAC increase in Japan.

We previously reported the mutation rate of SSBE, LSBE and EAC^30^. In order to more accurately evaluate the gene mutations in EAC and the surrounding BE, in this study, EAC and surrounding BE were collected from FFPE with LCM (as described in Supplementary Methods), and the number of cases was increased from previous study^30^. The genetic alterations identified in BE and EAC in the present study were less than those found in the previous study. The decrease in the mutation rate is considered to be due to an increase in cases. This study, which increased the number of cases, seems to show a more correct ratio. However, further case accumulation is desired to assess the true mutation rate.

Recently, several somatic mutations have been reported to be associated with EAC^9–16^, with *TP53* being the most frequent mutation^9,11–14,16,31^. In this study, as in western countries, the most common putative driver in the EAC groups was *TP53* (72.7%). This rate was comparable to previously reported rate of ≥ 70%^9,11–14,16,31^. In other words, the ratio of mutation for *TP53* in this study fitted into context with previous study including TCGA and ICGC data. This percentage was in accordance with the ratio of the overexpression-type mutation pattern of p53 staining (72.7%). However, other putative drivers known for SMG of EAC, *CDKN2A*^11,15^, *SMAD4*^31^ or *ARID1A*^32^ were not detected from the early EAC specimens of this study. We think the lack of finding was attributed to following factors. Weaver JMJ reported that for developing EAC, *TP53* is mutated firstly and *SMAD4* is recurrently mutated at the time of early invasive cancer^31^. The EAC in this study did not include any submucosal invasive cancer, and it is possible that EAC in this study was early cancers before *SMAD4* is mutated. Mutations might not be found due to the small number of EAC as well. Epigenetic silencing of *CDKN2A* often occurs in EAC^9^. In this cohort, though somatic mutation of *CDKN2A* was not identified, epigenetic silencing of *CDKN2A* might had occurred potentially. In summary, the genetic variants of *TP53* in Japanese early EAC were demonstrated to be similar to those in the West. On the other hand, the reason for the lack of mutation in *CDKN2A*, *SMAD4* or *ARID1A* need further examination.

A notion called *field defect* exists in EAC in which background BE mucosal defects are involved in the carcinogenesis. Recent studies from western countries have supported the notion. Agrawal et al.^11^ performed exome sequencing using frozen samples from EAC and BE adjacent to EAC and reported that most EAC mutations were already present in the surrounding BE. Another study by Ross-Innes et al.^15^ performed whole-genome and targeted sequencing using paired BE and EAC samples. They reported that BE in patients with EAC is highly mutated even in the absence of dysplasia. In this study, EAC and adjacent BE were pathologically judged by HE and separated by LCM. In other words, dysplasia was not observed in adjacent BE by HE staining. No significant difference exists in the number of putative drivers per lesion between the BE adjacent to EAC and EAC groups in the present study. Putative drivers were detected in six cases (66.7%) of the BE adjacent to EAC group in *TP53*. This percentage was comparable to the EAC group. These results may have also supported the notion of the field defect in BE.

*TP53* mutations are rarely detected in NDBE without concurrent dysplasia^33–35^. However, it is considered a progression risk if detected and Stachler MD, et al. reported that *TP53* mutations in BE tissues increased the adjusted risk of progression to high-grade dysplasia or EAC 13.8-fold (95% CI, 3.2–61.0)^35^. Recently, Ishikawa et al.^30^ reported that some BEs that fit only the Japanese diagnostic criteria may have a malignant potential to EAC. Surprisingly, the putative drivers of *TP53* in this study were detected in two (16.7%) of the 12 nondysplastic SSBE cases. However, they were not detected in LSBE cases. Furthermore, the number of putative drivers was not significantly different between the SSBE and LSBE groups. This is significant because such nondysplastic SSBE may have higher risk of progressing to high-grade dysplasia or EAC. The SSBE is generally considered to have less carcinogenic potential than the LSBE^36^. However, the risk per area of SSBE and LSBE may not be significantly different. These results alert the developing risk of EAC from SSBE in which attention should be given to the SSBE follow-up.

Known risk factors associated with EAC are white race, male sex, older age, hiatal hernia size, BE length, smoking, and high body mass index^37^. The detailed clinical information of the patients, including mentioned risk factors, and the endoscopic findings with the sequencing results were contrasted in an attempt to find an association. In this study, the only factor considered to be related to the number of putative drivers was age (≥ 65). Drinking habits also seemed to be a related factor. However, when limited to patients with EAC, it was found that there seemed to be no relationship. This may be because the EAC group had a higher percentage of drinkers than the NDBE group. Thus, these results may support that aging is a risk for progression of EAC, but other factors could not be shown in this study. On the other hand, this attempt is important for future EAC risk stratification, and further case accumulation is necessary.

The present study has several limitations. First, the present study was retrospective and used a relatively small number of patients in a single institution. Second, no other BE portions were examined because all biopsy samples were obtained from the most extended portion of the BE to avoid contamination from the stomach especially in patients with SSBE. Thus, a single biopsy tissue may not be sufficient to confirm the genetic information of the case because BEs are known to be heterogeneous and may have different mutations in different portions. A single biopsy might underestimate the potential of LSBE in developing EAC in this study. In other words, it is likely that the carcinogenic potential of LSBE has not been correctly assessed due to inappropriate biopsy site. This methodological flaw in this study was common in our previous report^30^.

In conclusion, the genetic variants of *TP53* in the Japanese early EAC were demonstrated to be similar to those in the West. Moreover, several *TP53* putative drivers exist in Japanese patients with nondysplastic SSBE. Thus, the risks of EAC should not be underestimated and appropriate follow-ups may be necessary even in patients with SSBE.

## Supplementary Information

Supplementary Information.

## Abbreviations

AF: Allele frequency
BE: Barrett’s esophagus
CLE: Columnar-lined epithelium
DNA: Deoxyribonucleic acid
EAC: Esophageal adenocarcinoma
EC: Esophageal cancer
EGJ: Esophagogastric junction
EMR-C: Endoscopic mucosal resection with cap
ESD: Endoscopic submucosal dissection
FFPE: Formalin-fixed, paraffin-embedded
LSBE: Long-segment Barrett’s esophagus
NDBE: Nondysplastic Barrett’s esophagus
NGS: Next-generation sequencing
PCR: Polymerase chain reaction
PV: Palisade vessel
SCJ: Squamous-columnar junction
SMG: Significantly mutated gene
SNP: Single-nucleotide polymorphism
SSBE: Short-segment Barrett’s esophagus
LCM: Laser capture microdissection
