# Supplementary material for: Genetic profiles of Barrett’s esophagus and esophageal adenocarcinoma in Japanese patients
Source: Sci Rep. 2021 Sep 3;11:17671. doi: 10.1038/s41598-021-97249-9 (PMC8417273; doi:10.1038/s41598-021-97249-9)
Supplement: Supplementary file 1 — Supplementary Information. [file 41598_2021_97249_MOESM1_ESM.docx]

**Supporting information legends**

**Supplementary Methods**

Supplementary Methods shows how to do deoxyribonucleic acid extraction, targeted next generation sequencing, and sequencing data analysis.

***Deoxyribonucleic acid extraction***

For EAC lesions, all resected and FFPE specimens were laser captured and microdissected for deoxyribonucleic acid (DNA) extraction. Lesional areas for microdissection were marked by pathologists on the guiding hematoxylin and eosine-stained slides to obtain EAC and adjacent BE in the tissue sections. Of the lesions, two had very few noncancerous BEs. Therefore, 11 EAC specimens were microdissected to 11 EAC areas and nine surrounding noncancerous BEs. DNA was extracted using the NucleoSpin® DNA FFPE XS kit (Takara Bio Inc., Tokyo, Japan) and was quantitated by fluorometry with the Invitrogen Qubit fluorometer and the Invitrogen Quant-iT double-strand DNA BR Assay Kit (Life Technologies, Carlsbad, CA, USA) as recommended by the manufacturers. All biopsy samples were quickly put into Allprotect Tissue Reagent (Qiagen, Hilden, Germany) and preserved at 4 °C until DNA extraction for NDBE. In addition, peripheral blood was obtained from all patients before or after endoscopy. Each buffy coat was isolated after centrifugation at 820×*g* at 4 °C for 10 min and stored at −80 °C until DNA extraction. The DNA of the tissue sample and the buffy coat of each patient was extracted with a QIAamp DNA Mini Kit (Qiagen). A NanoDrop (Thermo Fisher Scientific, Waltham, MA, USA) was then used to measure DNA concentration. All extracted DNA was stored at −80 °C.

***Targeted NGS***

Extracted DNA was amplified by multiplex polymerase chain reaction (PCR) with the premixed panel of this study and the HiFi Master Mix (Thermo Fisher Scientific). The amplicons were then treated with FuPa reagent (Thermo Fisher Scientific) to partially digest the primer sequences and phosphorylate the amplicons for optimization of the sequencing performance. The amplicons were then ligated to adapters with barcodes using an Ion Xpress Barcode Adapters kit (Thermo Fisher Scientific). The ligated library was purified using Agencourt AMPure XP reagents (Beckman Coulter, Brea, CA, USA), and the library concentration was quantified by real-time PCR with an Ion Library Quantitation Kit (Thermo Fisher Scientific). The concentration of each library was adjusted to 10 pM and was then enriched using emulsion PCR with Hi-Q™ View OT2 Kit (Thermo Fisher Scientific). After loading on an Ion 318 Chip (Thermo Fisher Scientific), sequencing was carried out on Ion PGM (Thermo Fisher Scientific). All procedures were performed following the manufacturer’s recommendations.

***NGS data analysis***

Sequence data was processed with Ion Torrent Suite Software. Raw signal data were analyzed using Torrent Suite version 5.16 after signal processing, base-calling adapter trimming, quality score assignment, PCR duplicate removal, read alignment to the reference human genome 19, quality control of mapping quality, and coverage analysis.

Nonsynonymous somatic and splice site mutations were identified by the Ion Reporter Server System (Thermo Fisher Scientific). In addition, buffy coat DNA was used as a reference to identify variants in EAC and NDBE. Mutations were filtered according to the following criteria to identify high-confidence somatic mutations: (1) the minimum count for mutant allele reads ≥5 in samples; (2) coverage depth ≥10 at the somatic variant site in samples; (3) high-confidence variant call (confident somatic variants = In), variant allele frequency (AF) ≥1% and *p* value cutoff 0.05; (4) variants present in the Single Nucleotide Polymorphism Database (dbSNP) version 138 are filtered out (UCSC common SNPs = Not In); and (5) no variant allele reads in the buffy coat. This analysis method was reported in a previous study, and it is believed that this data analysis is reasonable [22].

For each identified mutant gene, we analyzed whether it was oncogenic or not concerning OncoKB (http://oncokb.org/). In this study, oncogenic and likely oncogenic mutations were defined as putative drivers.
